# Network-based characterization and prediction of human DNA repair genes and pathways

**DOI:** 10.1038/srep45714

**Published:** 2017-04-03

**Authors:** Yan-Hui Li, Gai-Gai Zhang

**Affiliations:** 1 Institute of Cardiovascular Sciences and Key Laboratory of Molecular Cardiovascular Sciences, Ministry of Education, Peking University Health Science Center, Beijing, P. R. China; 2 Special Medical Ward (Geratology Department) First Hospital of Tsinghua University Beijing, P. R. China

## Abstract

Network biology is a useful strategy to understand cell’s functional organization. In this study, for the first time, we successfully introduced network approaches to study properties of human DNA repair genes. Compared with non-DNA repair genes, we found distinguishing features for DNA repair genes: (i) they tend to have higher degrees; (ii) they tend to be located at global network center; (iii) they tend to interact directly with each other. Based on these features, we developed the first algorithm to predict new DNA repair genes. We tested several machine-learning models and found that support vector machine with kernel function of radial basis function (RBF) achieve the best performance, with precision = 0.74 and area under curve (AUC) = 0.96. In the end, we applied the algorithm to predict new DNA repair genes and got 32 new candidates. Literature supporting four of the predictions was found. We believe the network approaches introduced here might open a new avenue to understand DNA repair genes and pathways. The suggested algorithm and the predicted genes might be helpful for scientists in the field.

Cellular DNA is subjected to continual attack by both reactive species inside cells and environmental agents such as ultraviolet light from the sun. DNA repair, which is an important biological process, maintains the integrity of DNA. To date, many DNA repair genes have been found^1^,^2^,^3^, and they are classified into eight specific DNA repair pathways: base excision repair (BER), mismatch excision repair, nucleotide excision repair (NER), homologous recombination repair, nonhomologous end-joining, direct reversal repair, DNA damage signaling (DDS) and translesion synthesis^1^. Traditional studies of DNA repair have focused primarily on searching for single genes or relatively simple pathways.

Proteins function not in isolation but through interaction with each other. With the accumulation of protein interaction data^4^, it has become possible to explore the network properties of interesting proteins. In fact, with network analysis, researchers have achieved great success in studying proteins encoded by human disease genes^5^,^6^,^7^. For example, Xu *et al*. studied five network features of proteins encoded by human disease genes and developed an algorithm to predict new ones^5^. Similarly, Goh KI *et al*. constructed a human disease network and revealed distinguishing features for proteins encoded by disease genes^6^. In a recent study, we analyzed network features of proteins encoded by *C. elegans* longevity genes and developed an algorithm to predict new candidates^7^. JB Brown *et al*. studied several general repair patterns exist in all organisms and developed an algorithm to annotate repair proteins in newly sequenced genomes^8^. To our knowledge, no work has been done to explore the network characteristics of Proteins encoded by DNA Repair genes (PDRs), and no algorithm has been developed to predict new DNA repair candidates.

In this work, we first downloaded protein interaction data from online predicted human interaction database (OPHID)^4^. Then, we compared three network features between PDRs and Proteins encoded by non-DNA Repair genes (non-PDRs) and found significant differences. Further analysis showed that proteins annotated to almost every DNA repair pathway have these network features. Finally, with the three features as inputs, we developed a support vector machine (SVM)-based algorithm to predict new DNA repair genes. 32 candidates were predicted. We searched the 32 predictions in PubMed, and found that four of them have been shown as DNA repair genes by recent researches.

## Results

### PDRs and DNA repair pathways tend to have more direct interaction proteins

**Figure 1:**
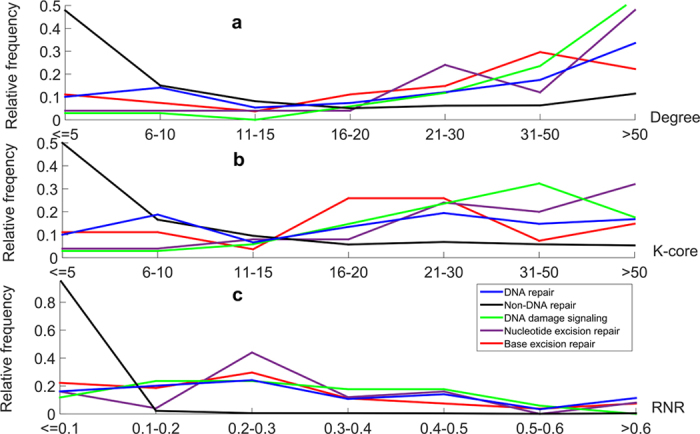
Distributions of degrees, *K*-cores and RNRs for PDRs, non-PDRs and proteins annotated to DNA repair pathways. PDRs and proteins annotated to DNA repair pathways tend to have higher average degrees, *K*-cores and RNRs than those of non-PDRs, respectively. The sum of the frequencies in different bins is 100% for PDRs, non-PDRs and proteins annotated to each DNA repair pathway. Repair Neighbor Ratio (RNR), Base excision repair (BER), DNA damage signaling (DDS) and nucleotide excision repair (NER). Two-sample Kolmogorov–Smirnov test was adopted.

The degree of a protein is defined as the number of its direct interaction proteins. From a network view, the higher degree a protein has, the more important that protein might be^9^. Based on protein interaction network downloaded from OPHID, we found that the average degree of PDRs is 54.71, whereas non-PDRs have an average degree of only 22.74, which is significantly lower than that of PDRs, *p* = 9.45E-25 by Kolmogorov–Smirnov test. (Table 1 and Fig. 1a). The proteins with the top three degrees are “TP53”, “PCNA” and “BRCA1” with degree = 543, 297 and 221, respectively.

**Figure 2:**
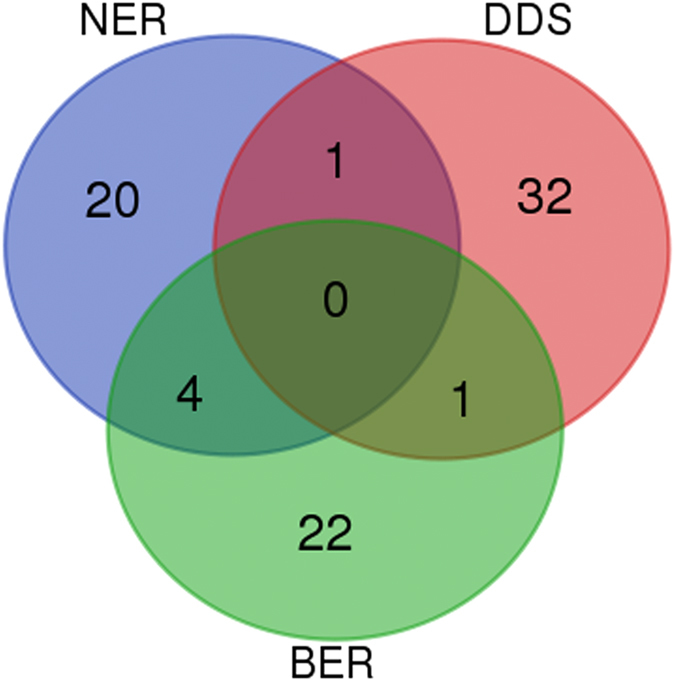
The number of shared proteins among BER, NER and DDS shown with a Venn diagram. Base excision repair (BER), DNA damage signaling (DDS) and nucleotide excision repair (NER).

In addition to PDRs, we wanted to evaluate the importance of DNA repair pathways. For each DNA repair pathway, we computed the average degree of the annotated proteins to represent its importance. We excluded DRR from analysis, since it has only three annotated proteins. As shown in Table 1, all DNA repair pathways except translesion synthesis have higher average degrees than that of non-PDRs. DDS has the highest average degree, 85.15, whereas translesion synthesis has the lowest, 8.5. The distributions of degrees for proteins of DDS, NER and BER can be found in Fig. 1a. They are the top three pathways with the largest number of annotations. The number of overlapping proteins among them were shown in Fig. 2, and symbols were listed in Supplementary Table S1.

### PDRs and DNA repair pathways tend to be located at the global network center

**Figure 3:**
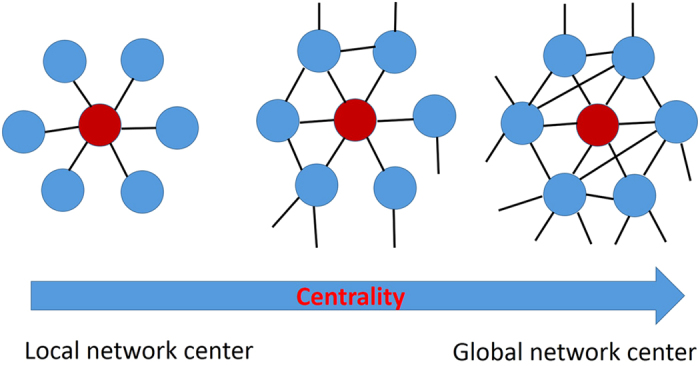
Local network center *v*ersus global network center. The centrality of a node is related to its degree and network neighborhood. A protein with high degree but low *K*-core value is defined local network center (left), while a node (not necessarily with very high degree) with high *K*-core value is defined global network center (right).

Proteins with high degrees might be at the global or local network center^10^. To distinguish the different locations, we computed another index called *K*-core. The *K*-core of a network can be obtained by recursively removing all nodes with a degree less than *K* until all nodes in the remaining network have a degree at least *K*. As shown in Fig. 3, proteins that with high degrees but low *K*-cores, are defined to be located at local network center^10^. In turn, proteins with high *K*-cores but are not necessarily with very high degrees are defined to be located at global network center^10^. The higher the *K*-core is for a protein, the more likely it is that the protein is located at the global network center. As shown in Table 1, the average *K*-core of PDRs is 26.09, which is significantly higher than the value of 11.91 for non-PDRs (*p* = 2.05E-22, Kolmogorov–Smirnov test). The distribution of *K*-cores for PDRs and non-PDRs can be found in Fig. 1b. The proteins with the top three *K*-core values are “PCNA”, “MMS19” and “TP53”, with *K*-core = 62, 62 and 60, respectively.

Similar to the degree analysis for repair pathways, we computed the average *K*-core for annotated proteins to reflect its centrality. As shown in Table 1, all DNA repair pathways except translesion synthesis have higher average *K*-cores than that of non-PDRs. Among the seven DNA repair pathways, mismatch excision repair has the highest average *K*-core, 39.20, whereas translesion synthesis has the lowest, 7.63. The distributions of *K*-cores for DDS, NER and BER can be found in Fig. 1b.

### PDRs and DNA repair pathways tend to directly interact with each other

To test whether PDRs tend to directly interact with each other, for each protein, we computed the Repair Neighbor Ratio (RNR), which is defined as the number of direct interaction proteins that belong to a PDR divided by its degree. For example, “PCNA” has 297 direct interaction proteins, 61 of which are PDRs. The RNR for “PCNA” is 0.2054 = 61/297. The average RNR of PDRs is 0.16, whereas non-PDRs have an average RNR of only 0.02, which is significantly lower than that of PDRs, *p* = 4.69E-126 (Kolmogorov–Smirnov test Table 1 and Fig. 1c).

Similarly, for each repair pathway, we computed the average RNR of the proteins annotated to it. As shown in Table 1, all repair pathways have a higher average RNR than that of non-PDRs. Among the seven repair pathways, translesion synthesis has the highest average RNR, 0.44. The distributions of RNR for DDS, NER and BER can be found in Fig. 1c.

### An algorithm to predict new DNA repair candidates

As analyzed above, the network features of PDRs are significantly different from those of non-PDRs. We reasoned that these features could be used for predicting new DNA repair candidates. SVM was employed as the classifier. Five-fold cross-validation was used to evaluate classifier performance. SVM with a radial basis function as the kernel function was found to provide the best performance, with *precision* = 0.74, *recall* = 0.52, *F1* = 0.60, and *AUC* = 0.96. The performance measures of SVM with a poly kernel function and decision tree classifier were not as good (see Table 2).

The trained classifier was used to predict new candidates. 32 new ones were predicted. The gene symbols and network features can be found in Table 3. We searched the top ten predicted genes in PubMed. Recent researches have shown four of them as DNA repair genes (Table 4). For example, *fan1* was predicted to be a DNA repair gene with a posterior probability 0.99. Recent work reported that DNA interstrand cross-links can be repaired by the Fanconi anemia pathway and through FA-independent processes involving the FAN1 nuclease^11^. Another example, *pif1*, was predicted with a posterior probability 0.99. It has been shown that break-induced replication requires DNA damage-induced phosphorylation of Pif1^12^.

Because the network features of the proteins annotated to DDS, NER and BER are different from those of non-PDRs, an obvious question is whether it is possible to directly predict proteins to a DNA repair pathway. Thus, we defined proteins annotated to DDS (NER or BER) as positive examples, and the remaining ones in the network as negatives. Similarly, SVM was used as the classifier. Unfortunately, the performance measures were poor, with *AUC* = 0.51, 0.53, and 0.53 for DDS, NER and BER, respectively. This might because there were too few positive samples.

## Discussions

In this work, for the first time, we successfully introduced network biology to study properties of PDRs. We found that PDRs tend to be higher in degrees, *K*-cores, and RNRs. These findings are consistent with their functional importance. The network approaches introduced here might open a new avenue to study PDRs and DNA repair pathways. Based on these features, we developed the first algorithm to predict new PDRs. The support vector machine with kernel function of radial basis function (RBF) achieve the best performance. Unfortunately, our current algorithm could not accurately predict new genes to specific DNA repair pathway as mentioned in the results. This might trigger readers to try new models or features to further improve the prediction performance. In the end, the algorithm predicted 32 new candidates. And literature supporting four of the predictions were found. We think that both the algorithm and the predictions might be helpful to scientists in the field.

PDRs were classified into eight different pathways. We analyzed network features for both all PDRs and PDRs annotated to each DNA repair pathway. On one hand, we wanted to show that PDRs annotated to almost every repair pathway instead of only some pathways have these network features. On the other hand, we wanted to predict new candidates directly to each DNA repair pathway. As shown in the results, we trained the classifier using both the combined and separated PDRs of every DNA repair pathway. The performance of the classifier for the combined PDRs is excellent. However, the performance measures are poor for the separated ones. This might because the number of positives is too few for the separated pathway, which covered up by large number of negatives.

To characterize DNA repair genes and pathways, a reliable list of DNA repair genes and pathways is very important. Different researchers might group DNA repair genes to different pathways based on their knowledge. However, we do not want to both define the DNA repair genes and pathways, and develop an algorithm to analyze them by ourselves, because readers would doubt whether the good performance of the algorithm is due to the definitions. Thus, we used the DNA repair genes and pathways provided and defined by repairtoire^1^. Though repairtoire might have defects like that some newly discovered DNA repair genes might not be collected and the definitions of DNA repair pathways are not reasonable to everyone, it is an independent dataset to our algorithm.

OPHID^4^ dataset of protein interactions was used for network topological analysis in this work. Though it is the largest dataset of protein interactions, it covers only a part of all interactions in human. Thus, some limitations are inevitable. For example, the more a protein is studied, the more likely the protein has higher degree. *K*-core and RNR are indirectly and less affected by such research bias. To know whether the results found in this work is data-dependent, we computed these network indexes using another dataset of protein interactions downloaded from human protein reference database^13^, which covers 9, 453 proteins and 36, 867 edges. We found that the average degree, *K*-core and RNR for PDRs are 13.75, 5.91 and 0.44 respectively, significantly higher than 7.80, 4.07 and 0.02 for non-PDRs. These results are consistent with the results computed based on OPHID dataset.

Previous studies have shown that proteins encoded by human disease genes and aging genes tend to be higher in network degree, to locate at the network center and to interact directly with each other^5^,^7^. Considering the network features found in this work for PDRs, it would be interesting to explore common network features between proteins encoded by human disease genes, aging genes and DNA repair genes. Recently, an algorithm was suggested for classifying DNA repair genes into aging-related versus non-aging-related based on gene functional categories and evolutionary changes^14^. We think integrating network features into the input of the classifiers would improve such classification.

We defined 149 PDRs as positive samples and the remaining ones (14,557 = 14,706 -149) in the network as negative samples. Because there may be true PDRs among the negative samples, the classifiers tend to be underestimated, leading to false negatives. Recently, some researchers defined their negative samples by randomly choosing the same number of proteins as in their positive samples from the genome^5^,^15^. One weakness of this strategy is that there might be sampling bias resulting in bias in classification evaluation. Another weakness, as discussed by Chad L Myers^16^, is that users should take care to interpret the measures of such a classification because they are correct only under the assumption that the ratio of positive to negative examples in the application domain is also 1:1.

## Material and Methods

### Data source

A protein interaction dataset consisting of 14,801 proteins and 310,570 edges was downloaded from OPHID (http://ophid.utoronto.ca/ophidv2.204/)^4^. After deleting self-interactions and redundant interactions, we obtained a final network including a total of 14,706 proteins and 169,560 edges. A list of 154 human DNA repair genes was downloaded from repairtoire (http://repairtoire.genesilico.pl/)^1^. They were mapped to protein identifiers by HUGO gene nomenclature committee^17^. 149 PDRs are covered by the OPHID network. According to repairtoire, DNA repair genes can be classified into eight specific repair pathways. The number of PDRs for each pathway can be found in Table 1.

### Network topological features

Three network features, i.e., degree, *K*-core and RNR, were analyzed in this work. The definitions for them can be found in Table 5. They were computed by an R package, igraph^18^.

### Kolmogorov–Smirnov test

In statistics, the two-sample Kolmogorov–Smirnov test is one of the most useful nonparametric methods for comparing two samples. It is sensitive to differences in both the location and shape of the empirical cumulative distribution functions of the two samples.

### Classifier

SVM was used as the classification model in this work. The software LIBSVM 3.20^19^ was employed, in which a radial basis function was chosen as the kernel function. The default values of parameters *c* and *g* were used. According to LIBSVM, the values of the three network features were first scaled to [−1, 1] and then used as inputs.

For a given test example x, an SVM classifier outputs a predictive value that represents the distance of x from the optimal separating hyperplane in the feature space. The sign of this predictive value indicates the class j to which example x belongs, where j ∈ {+1, −1}. However, knowing the class label (+1, or −1) or the predictive value is not sufficient to evaluate a classification. A binning technique was used to convert predictive values to posterior probabilities^20^, which has been implemented internally in the LIB-SVM software package. The posterior probability ranges from 0 to 1. The larger the posterior probability is for a protein, the more likely it is that the protein is a PDR.

### Positive and negative samples

The 149 PDRs obtained from repairtoire^1^ were defined as positive samples. All remaining proteins (14,557 = 14,706-149) in the network were defined as negative samples. The negative samples are highly likely to have unknown PDRs, thus the performance of classifiers tends to be underestimated.

### Classifier evaluation

To evaluate the performance of SVM, 5-fold cross validation was adopted. In each round, 20 percent of the samples were left out as the test set, and the remaining were used as the training set. As in previous works^7^,^21^, *precision, recall* and *F1* were used to evaluate the classifiers. Of the proteins predicted as PDRs, the numbers of true positives (*TP*) and false negatives (*FN*) were counted. Of the proteins predicted as non-PDRs, the numbers of true negatives (*TN*) and false positives (*FP*) were also counted. Then, the *precision, recall* and *F1* scores were calculated as follow (equations 1, 2, 3).


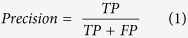



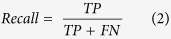



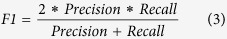


*Precision* is the fraction of true positives among the predicted positives, and *recall* is the fraction of gold standard positives that are predicted as true positives. *F1* is used to evaluate the overall performance of a classifier. Receiver operating characteristic curves are another measure that is often used to evaluate classifiers, thus, we also computed area under curve (*AUC*) in this work (Table 2).

## Conclusions

For the first time, we successfully introduced network biology to study properties of PDRs. We found that PDRs tend to be higher in degrees, *K*-cores, and RNRs. Based on these features, we developed the first algorithm to predict new PDRs. The efficient algorithm predicted 32 new DNA repair candidates.

## Additional Information

**How to cite this article:** Li, Y.-H. and Zhang, G.-G. Network-based characterization and prediction of human DNA repair genes and pathways. *Sci. Rep.* **7**, 45714; doi: 10.1038/srep45714 (2017).

**Publisher's note:** Springer Nature remains neutral with regard to jurisdictional claims in published maps and institutional affiliations.

## Supplementary Material

Supplementary Table S1

## Figures and Tables

**Table 1:** Network features of DNA repair pathways.

Class	Size	Degree	*K*-core	RNR
DNA damage signaling	34	85.15	34.68	0.27
Nonhomologous end-joining	11	72.73	31.18	0.32
Mismatch excision repair	10	77.9	39.20	0.26
Nucleotide excision repair	25	75.76	35.32	0.32
Homologous recombination repair	21	52.48	25.00	0.38
Base excision repair	27	49.67	24.78	0.25
Translesion synthesis	8	8.50	7.63	0.44
Direct reversal repair	1	—	—	—
DNA repair	149	54.71	26.09	0.16
Non-DNA repair	14, 557	22.74	11.91	0.02

The degree of a protein is the number of its direct interaction proteins. The RNR is the ratio of the number of direct interaction proteins that belong to PDRs to its degree. The *K*-core of a network can be obtained by recursively removing all nodes with degrees less than *K* until all nodes in the remaining network have degrees at least *K*.

**Table 2:** Performances of different classifiers at distinguishing PDRs from non-PDRs.

Classifier	Precision	Recall	F1	AUC
SVM (polynomial)	0.71	0.44	0.52	0.93
SVM (radial basis function)	0.74	0.52	0.60	0.96
Decision Tree	0.56	0.51	0.53	0.76

*Precision* is the fraction of true positives among the predicted positives, whereas *recall* is the fraction of gold standard positives that are predicted as true positives. *F1* was used to evaluate the overall performance of a classifier. *AUC*: area under curve.

**Table 3:** The 32 predicted DNA repair genes.

UniProtID	Symbol	Degree	*K*-core	RNR	Posterior probability
Q13472	TOP3A	53	36	0.3396	0.9986
P49642	PRIM1	55	44	0.2909	0.9982
Q9Y2M0	FAN1	16	15	0.6250	0.9935
Q9H611	PIF1	32	27	0.3438	0.9864
P51530	DNA2	36	32	0.2778	0.9706
Q8NEM0	MCPH1	18	17	0.4444	0.9588
Q12888	TP53BP1	43	26	0.3023	0.9430
Q9BX63	BRIP1	9	9	0.7778	0.9405
Q14565	DMC1	30	26	0.3000	0.9402
Q86WJ1	CHD1L	25	18	0.4000	0.9371
Q9HAW4	CLSPN	19	16	0.4211	0.9125
P09884	POLA1	69	49	0.1739	0.8667
Q8N2Z9	APITD1	13	9	0.6154	0.8498
Q9H967	WDR76	29	28	0.2414	0.8106
Q8IW19	APLF	8	7	0.7500	0.8034
Q14527	HLTF	67	44	0.1642	0.7396
Q96S55	WRNIP1	43	31	0.2093	0.7337
Q6P1K8	GTF2H2C/GTF2H2C_2	33	30	0.2121	0.7124
Q13156	RPA4	7	6	1.0000	0.6861
Q0VG06	FAAP100	8	7	0.6250	0.6675
Q13112	CHAF1B	48	39	0.1667	0.6444
Q99728	BARD1	98	33	0.1837	0.6327
A8MT69	STRA13	10	9	0.5000	0.6280
P49760	CLK2	10	9	0.5000	0.6280
Q8WVB6	CHTF18	15	12	0.4000	0.5979
Q16658	FSCN1	54	37	0.1667	0.5931
Q6PCD5	RFWD3	14	14	0.3571	0.5925
P49643	PRIM2	42	37	0.1667	0.5874
O75792	RNASEH2A	25	23	0.2400	0.5696
Q9NYB0	TERF2IP	19	16	0.3158	0.5543
Q9Y294	ASF1A	184	59	0.1250	0.5185
Q9H9A7	RMI1	14	10	0.4286	0.5090

The degree of a protein is the number of its direct interaction proteins. The RNR of a protein is the ratio of the number of its direct interaction proteins that belong to PDRs to its degree. A *K*-core of a network can be obtained by recursively removing all nodes with degrees less than *K* until all nodes in the remaining network have degrees at least *K*. “Posterior probability” was outputted by SVM to reflect the reliability of the prediction. A link to NCBI was provided for each gene symbol.

**Table 4:** Literature supporting the predictions.

Symbol	Posterior probability	PMID	Description
FAN1	0.99	26052075	Germline Mutations in FAN1 Cause Hereditary Colorectal Cancer by Impairing DNA Repair
FAN1	0.99	25430771	DNA interstrand cross-links can be repaired by the Fanconi anemia pathway and through FA-independent processes involving the FAN1 nuclease.
PIF1	0.99	25329304	Break-induced replication requires DNA damage-induced phosphorylation of Pif1
DNA2	0.97	26420828	DNA2-mediated resection is a major mechanism for the repair of DSBs with 5′ adducts
TP53BP1	0.94	24326623	53BP1 promotes non-homologous end-joining-mediated DSB repair while preventing homologous recombination

“Posterior probability” was outputted by SVM to reflect the reliability of the prediction. “Descriptions” were obtained from the literature.

**Table 5:** Formal representation of graph measures.

Name	Function	Description
Degree	*K*_*i*_	The number of interaction proteins of node *i*
RNR	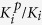	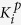 is the number of links between node *i* and PDRs
*K*-core	*K*	A *K*-core of a graph can be obtained by recursively removing all nodes with a degree less than *K* until all nodes in the remaining graph have a degree at least *K*.

The functions are the definitions of the network topological features. The descriptions give explanations for the symbols in the definitions.
