# Supplementary material for: Network-based characterization and prediction of human DNA repair genes and pathways
Source: Sci Rep. 2017 Apr 3;7:45714. doi: 10.1038/srep45714 (PMC5377940; doi:10.1038/srep45714)
Supplement: Supplementary Table S1 [file srep45714-s1.pdf]

# ONLINE SUPPLEMENTARY DATA

## Network-based characterization and prediction of human DNA repair genes and pathways

Yan-Hui Li[1,*] and Gai-Gai Zhang[2]

[1] *Peking University Health Science Center, Beijing, P. R. China*

[2]*Special Medical Ward (Geratology Department), First Hospital of Tsinghua University Beijing, P. R. China*

**Supplementary Table S1. The shared genes of the eight DNA repair pathways**

| DNA Repair Pathway | #Gene | Symbol |
|---|---|---|
| BER MMR NER | 2 | POLD1 POLE |
| DDS NER | 1 | RFC1 |
| BER NER | 2 | PCNA LIG1 |
| NER NHEJ | 1 | XPF |
| BER DDS | 1 | HUS1 |
| DDS HRR | 5 | BRCA1 FANCC MRE11A FANCA BLM |
| BER NHEJ | 2 | RECQL2 POLL |
| BER TLS | 1 | POLH |
| NHEJ TLS | 1 | POLM |
| NER | 19 | MMS19 CUL4A GTF2H5 XPA CSB XPB DDB1 XPD XPC RPA1 DDB2 XPG RAD23B ERCC1 GTF2H2 GTF2H3 CSA GTF2H1 GTF2H4 |
| DDS | 27 | RFC3 COPS5 RAD23A RAD50 RFC5 CDK7 CHEK1 NBN CHEK2 DCLRE1B TP53 MNAT1 GPS1 MDC1 RAD1 RAD9A TOPBP1 RAD18 ATR CDKN1A RFC2 ATRIP CCNH RFC4 RAD17 ATM DCLRE1A |
| BER | 19 | MPG PARP2 TDG APTX NTH MBD4 MUTYH UNG PNKP POLB XRCC1 NEIL1 NEIL2 OGG1 APEX1 FEN1 SMUG1 PARP1 APEX2 |
| MMR | 8 | EXO1 MLH1 MLH3 MSH2 MSH3 PMS2 MSH6 PMS1 |
| HRR | 16 | RAD52 FANCE MSH5 MSH4 FANCL FANCB EME1 EME2 RAD51 FANCG MUS81 FANCI FANCD2 BRCA2 C19ORF40 FANCF |
| DRR | 1 | ALKBH2 |
| NHEJ | 7 | LIG4 NHEJ1 Ku80 DCLRE1C PRKDC Ku70 XRCC4 |
| TLS | 6 | REV3L POLQ POLN POLK REV1 POLI |

Note: base excision repair (BER), mismatch excision repair (MRR), nucleotide excision repair (NER), homologous recombination repair (HRR), nonhomologous end-joining (NHEJ), direct reversal repair (DRR), DNA damage signaling (DDS) and translesion synthesis (TLS).
